# Relative risk of childhood and adolescence cancer in Iran: spatiotemporal analysis from 1999 to 2016

**DOI:** 10.1186/s13104-023-06629-z

**Published:** 2024-01-18

**Authors:** Hasti Hashemi, Behzad Mahaki, Rahman Farnoosh

**Affiliations:** 1 Department of Statistics, Science and Research Branch, Islamic Azad University, Tehran, Iran; 2 Department of Biostatistics, School of Health, Kermanshah University of Medical Sciences, Kermanshah, Iran; 3 School of Mathematics, Iran University of Science and Technology, Tehran, Iran

**Keywords:** Adolescents, Children, Disease mapping, Geospatial, Neoplasms

## Abstract

**Objective:**

Cancer is the third leading cause of death in the world with increasing trends in Iran. The study of epidemiology, trend, and geospatial distribution of pediatric cancers provides important information for screening as well as early detection of cancer and policy making. We aimed to assess the spatio-temporal disparity of childhood and adolescence cancer risk among provinces of Iran.

**Methods:**

In this retrospective study, we estimated geospatial relative risk (RR) of childhood cancer in provinces of Iran using data from 29198 cases. We used BYM and its extended spatiotemporal model in Bayesian setting. This hierarchical model takes spatial and temporal effects into account in the incidence rate estimation simultaneously.

**Results:**

The relative risk of cancer was > 1 for 45% of the provinces, where 27% of provinces had significantly ascending trend. North Khorasan, Yazd and Qazvin provinces had the highest risk rates while Sistan-Baluchistan province showed the lowest risk of cancer. However, the differential trends was highest in Sistan-Baluchistan, Bushehr, Hormozgan, and Kohgilouyeh-Boyerahmad. Both the point estimate and the trend of risk was high in Tehran.

**Conclusion:**

The geographic pattern and trend of cancer in children seems to be different from that in adults that urges further studies. This could lead to increased health system capacity and facilitate the access to effective detection, research, care and treatment of childhood cancer.

## Background

Cancer is the second leading cause of death in children worldwide [1]. According to the estimates, about 400,000 children and adolescents 0–19 years of age develop cancer [2]. neoplasms are the main cause of death among non-communicable diseases in children of 5–14 years old in the world [3]. Age-standardized incidence rates (ASIR) show a positive correlation between cancer incidence and human development index. ASIR is the highest (182 per million) in high-income countries, whereas the age-standardized mortality rates (ASMR) is in reverse direction with the highest rates in low- and middle-income countries [4, 5]. ASR in boys is 163.2 and 151.4 per million in general and pediatric datasets which is higher than that in girls with corresponding values of 143.6 and 129.4 [6]. Data from population-based registries indicate higher ASR in age groups of 15–19 and 0–4 than ASR in other age groups [6]. In west Asia, where Iran is located, incidence rates are 140.9 and 150.7 per million for age groups 0–14 and 0–19, respectively [6].

Various factors ranging from the use of advanced diagnostic modalities to the supportive care has led to the improvements in the 5-year survival rate of > 80% in high-income countries, whereas the conditions is remarkably worse in low- and middle-income countries, where almost 90% of the patients reside [5, 7]. Cancer incidence among children and adolescents is lower compared to adults. However, it may cause more severe consequences with higher recurrence rate and therapeutic complications [8, 9]. Furthermore, childhood cancer cannot generally be prevented or identified through screening and the increases in the burden of non-communicable disease, such as cancer, has attracted the global focus towards these diseases [5, 6, 10].

Estimates of childhood cancer incidence rates in Iran were increasing from 10.1 in 1990 to 11.9 (per 100,000) in 2016 suggesting 0.64 of annual percent change with higher incidence and increase in males [3]. Recent reports indicate increasing trends ASR with annual percent change of 1.6% in females and 2.1% in males during 1990–2016 [3, 11]. However, as the incidence of cancer depends highly in geographically distributed conditions such as genetic and economic factors, the use of spatial and spatio-temporal methods could be effective in the identification of high-risk areas, development of new hypotheses, conducting focused research, and optimal allocation of resources. Determination of changes in cancer risk in terms of geographical factors can identify the role of environmental carcinogenic, genetic and race/ethnical factors [12]. Moreover, reviewing the published reports shows that childhood cancer research is undervalued in the world, especially in low- and middle-income countries [13]. The analysis of trends could also improve understanding of etiology and healthcare disparities [14]. Nevertheless, no study in Iran has addressed the issue of neighboring effect in estimates of childhood cancer risk. Hence, the aim of the present study was to estimate the spatiotemporal risk of cancer in Iranian children from 1999 to 2016. The results would include such neighboring effects in the estimates and provide a basis for constructing more focused and precise hypotheses on cancer incidence and assist in budget allocation.

## Methods

The data on incident cancer cases was obtained from the Deputy of Cancer and Non-communicable Diseases Center of Iran Ministry of Health and Medical Education (MOHME). National cancer registry (NCR) was established in 1984 and has a local office in each province and is responsible for data collection and management [15]. In 1993, MOHME initiated a cancer registry program through grants to the Cancer Institute to develop regional population-based cancer registries [16]. New cases of cancer are recorded in provincial organizations and annually reported to the central deputy. These data are synchronized, validated and distributed annually, although with a delay of 4 years [15]. We included all confirmed cancer cases of with age < 15 years in both genders in the analysis. We analyzed data in terms of year and province from 1999 to 2016. The cancer cases were coded according to their topography and morphology defined by international classification of cancers (ICD-O-3/WHO 2008).

To circumvent the problems of common ASR estimates of the annual risk, Besag, York and Mollie (BYM) suggested a Poisson distribution for observed counts in each region such that the average occurrence rate parameter is a scaled value of expected values [17–19]. Bernardinelli et al. proposed a similar model capable of modeling trends over time [20]. This model estimates the risk in a region by considering the prior distribution of risk in neighboring regions as well as temporal trends as follows. Let *O*_ik_ be the number of patients in the *i-*th region or province with total population of *n*_ik_ at time *k*. Then, the expected number of patients for the *i*-th region at time *k*, *E*_ik_, would be

$${E_{ik}} = {n_{ik}}\left( {{{\sum\limits_{i = 1}^P {{O_{ik}}} } \mathord{\left/ {\vphantom {{\sum\limits_{i = 1}^P {{O_{ik}}} } {\sum\limits_{i = 1}^P {{n_{ik}}} }}} \right. \kern-\nulldelimiterspace} {\sum\limits_{i = 1}^P {{n_{ik}}} }}} \right),$$

where *P* is the total number of regions. Then the observed frequencies is modeled as

$${O_{{\text{ik}}}} \sim {\text{Poisson}}\left( {{E_{{\text{ik}}}}R{R_{{\text{ik}}}}} \right),$$

$$\log \left( {R{R_{{\text{ik}}}}} \right) = \alpha + \log {E_{{\text{ik}}}} + {u_{\text{i}}} + {v_{\text{i}}} + \beta .{t_E} + {\delta _{\text{i}}}.{t_{\text{k}}},$$

in which *ν*_i_ and *u*_i_ are correlated and non-correlated heterogeneity indices for each region, respectively. *β* denotes the average time effect and *δ*_i_ stands for province-specific time effect called differential trend (DT) that represents the incidence acceleration/deceleration in each province relative to the country-wide average. Sum of overall time effect and DT shows the temporal trends (TT) for each province. Actually, DT and TT provide relative and total time effects for each province, respectively. It is supposed that *u*_i_ has a conditional auto-regressive (CAR) distribution defined as:

$$\left[ {{u_i}|{u_j},i \ne j,{\tau_u^2}} \right] \sim N\left( {{{\bar u}_i},{\tau_i^2}} \right),$$

in which the effect for each region *i* has normal distribution with harmonic means on the neighboring regions defined as follows:

$${\bar u_i} = \frac{{\sum\limits_j {{u_j}{w_{ij}}} }}{{\sum\limits_j {{w_{ij}}} }},$$

where, j represents neighboring regions of the area *i*. The weight is always considered as 1 for neighboring and 0 for other regions. The total variance is defined as

$${\tau_i^2} = \frac{{\tau_u^2}}{{\sum\nolimits_j {{w_{ij}}} }}.$$

For u_i_, we set normal prior distribution for mean and inverse-gamma prior distribution for the variance. Bayesian post probability was also employed for calculating *p*-values for testing the null hypothesis of RR = 1 using Markov chain Monte Carlo method. Gelman-Rubin diagrams were used to assess model convergence and performance. Model selection was based on DIC criterion. All analyses were implemented in OpenBUGS 3.2.2 software and the results were plotted in Arc GIS [21].

The protocol of the study was approved by the Ethics Committee of Islamic Azad University (code: 123480793474175162492227). Due to the non-intervnetional, retrospective nature of the study and the use of aggregate data, obtaining consent to participate was waived by the Ethics Committee of Islamic Azad University.

## Results

Iran had almost a total population of 23 million people under the age of 18 in 2016. In total, out of 93,274 incident pediatric cancer cases recorded in 17-year period under study, 20,043 cases (68.46%) were male and 9155 cases (31.35%) were female. Standardized cancer incidence increased from 129.2 per million in 1999 to 132.3 per million in 2016 (IRR = 0.976, *p*-value = 0.355).

**Fig. 1:**
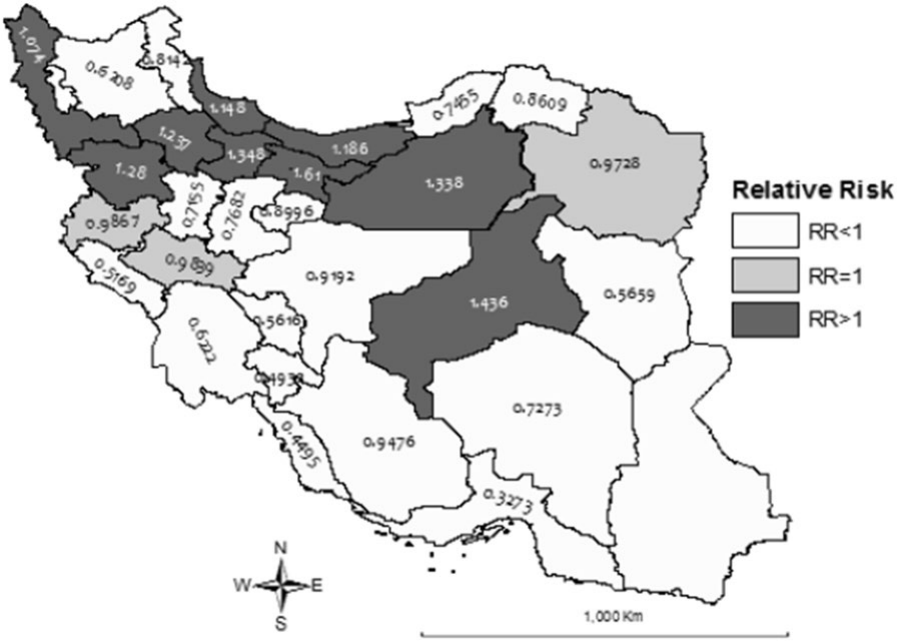
17-year cumulative risk of cancer in Iranian children based on BYM model

The results of BYM model on cumulative spatial risk of cancer in different provinces of the country is shown in Fig. 1. According to the estimates, the cumulative risk range was from 0.18 in Sistan-Baluchistan to 1.61 in Tehran (including Alborz) with estimated RR > 1 in 9 provinces.

**Fig. 2:**
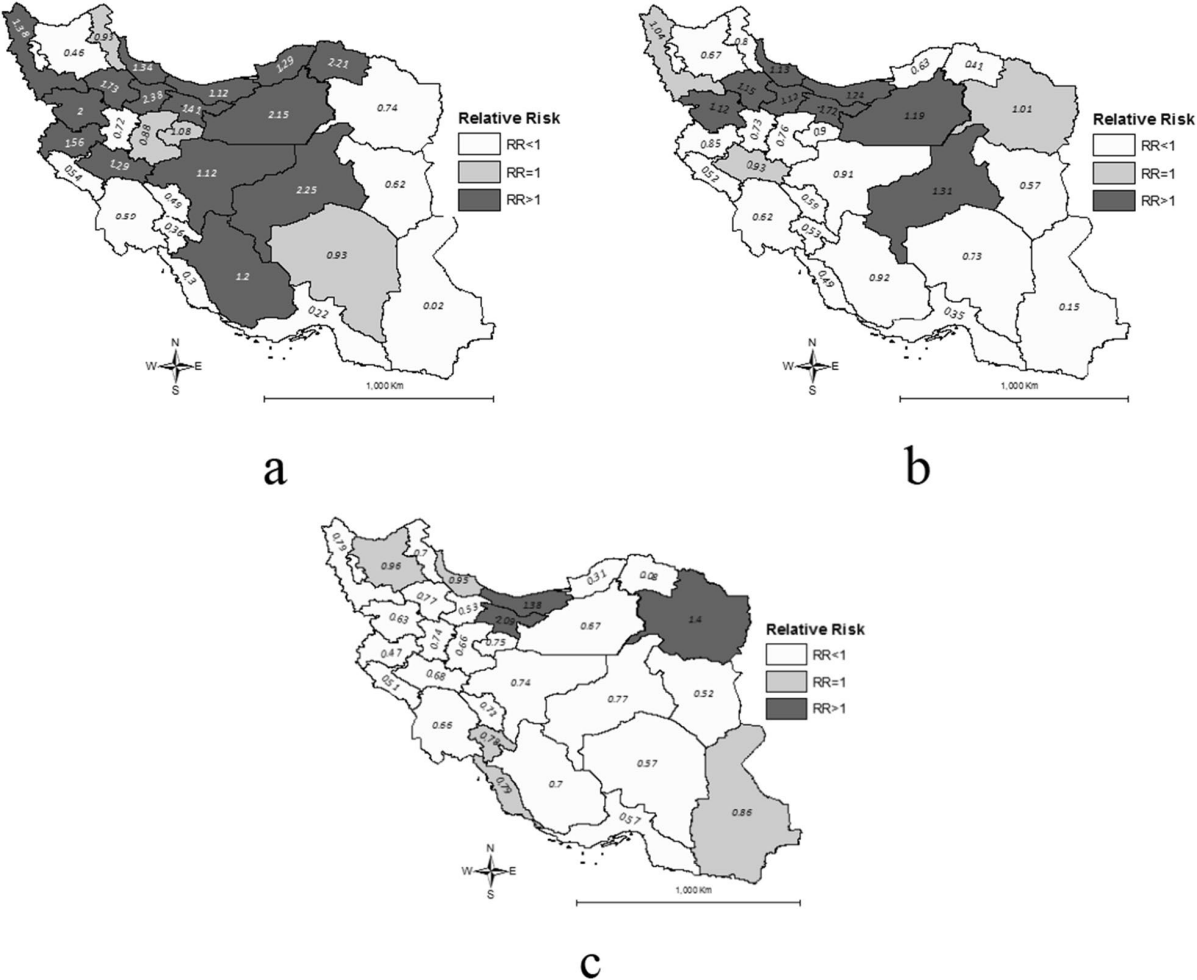
Bayesian estimates of spatial risk of cancer in Iranian children and adolescents in 2001 (**a**), 2004 (**b**) and 2016 (**c**)

Point estimates of cancer incidence in Iranian children in 2001, 2004 and 2016 using BYM model are mapped in Fig. 2. This figure suggests increasing risk trends in eastern and southern provinces.

**Fig. 3:**
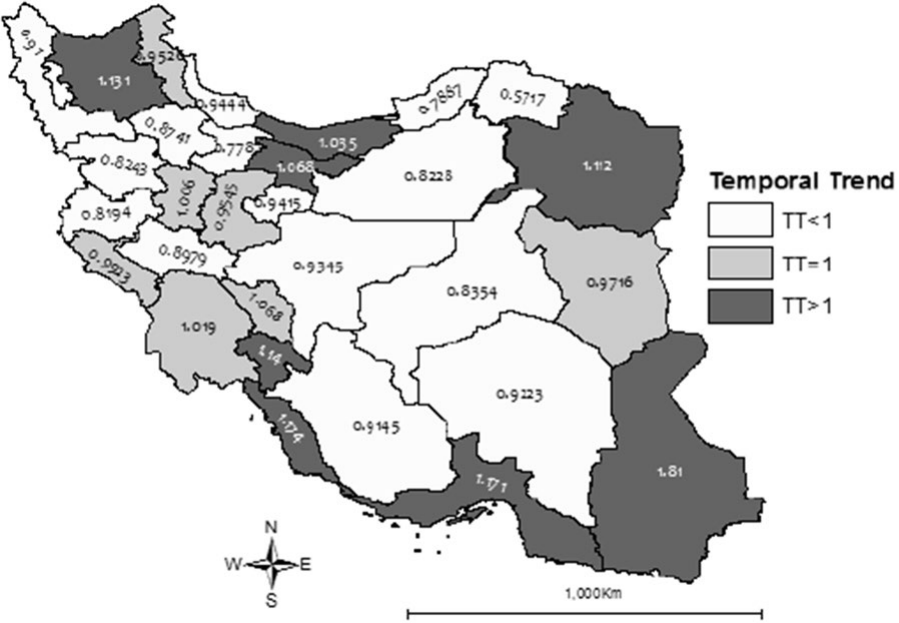
Temporal trends of cancer among Iranian children and adolescents from 1999 to 2016

**Fig. 4:**
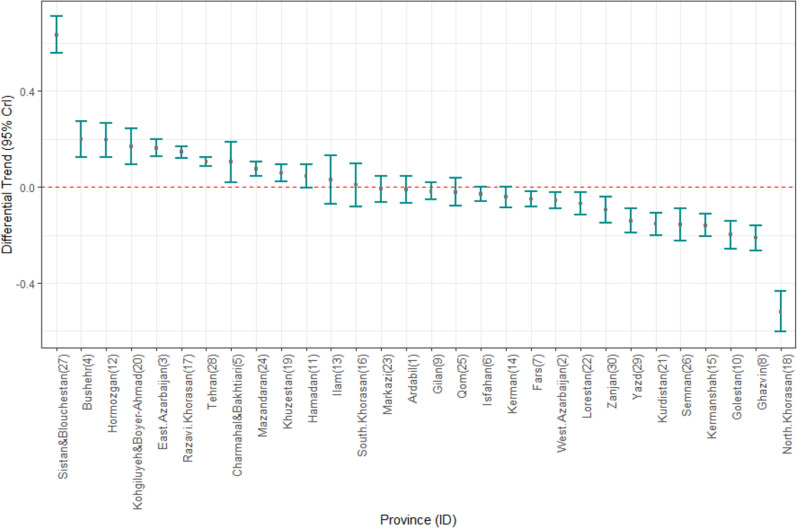
Differential trend of cancer incidence in Iranian children from 1999 to 2016

To further investigate the risk pattern, we plotted temporal trends (TT) and differential trends (DT) in Figs. 3, 4. It is clear from Fig. 3 that the incidence trends were increasing in 8 provinces (26.66% of provinces) where Sistan-Baluchistan with TT = 1.81 (95% credible interval: [1.67, 1.96]) had the sharpest increase in the incidence of childhood cancer in the country.

Differential trends shown in Fig. 4, compares trends of each province to the average country-wide trend in study period. Again, Sistan-Baluchistan has the sharpest increasing relative trend in the country followed by Bushehr, Hormozgan, and Kohgilouyeh-Boyerahmad provinces, where the slope of the trend is the smallest for North Khorasan.

## Discussion

In the current study, we addressed the spatiotemporal epidemiology of childhood cancer in Iranian children. Our results indicate the highest risk of cancer in northern parts of Iran and Yazd province. However, the trends is ascending in non-central regions with the sharpest increase in TT and DT in Sistan-Baluchestan in southeast. Cumulative cancer risk was estimated 87.34 per million among children younger than 18. Previous studies in Iran reported childhood cancer incidence as 48–144 per million with higher rates in male children [22].

Cancer incidence in children and adolescents is increasing worldwide [6, 23–27]. The incidence is affected by various factors such as sex, age, race, and is characterized by geographical distribution where the majority of cases occur in low- and middle-income countries [22, 28, 29]. This pattern is also present in survival and mortality rates [2, 6]. The burden of disease is highest in high-income countries and the lowest in low- and middle-income countries [5]. All this underpins that besides baseline and genetic factors, the disease incidence and outcome is geographically distributed according to environmental factors and economic status [30]. Spatiotemporal analysis is a useful tool in screening regional disparities in disease incidence and mortality [31]. Due to cost-effectiveness barriers to childhood cancer screening, the knowledge on spatial and temporal trends of cancer incidence can provoke provincial cancer registry officials to monitor and revise detection and registration standards and help planners to invent balanced care programs [3, 32].

Various spatiotemporal studies have been reported in Iran in different disciplines ranging from cancer to tuberculosis [17, 18, 33–35]. Cancer studies show a different geographical distribution and temporal patterns for different cancer sites. The highest estimated risk of bladder cancer in the adults is in Gilan and Semnan, where risk of breast cancer is highest in Tehran and Isfahan [33, 35]. Mahaki et al. reported northern, north western and eastern provinces as the high-risk provinces regarding esophagus cancer [34].

Our findings suggests the highest overall risk of childhood cancer incidence in central and northern provinces (North Khorasan, Qazvin, Yazd and Semnan) while the eastern and southeastern provinces exhibited lower risks. However, the trend maps tell a different story. Three selected annual incidence maps, as well as temporal trend estimates show a rapid increasing trend for eastern and southern provinces along with East Azerbaijan,‌ Tehran and Mazandaran. There could be various justifications. Part of, but not whole, this increase could be attributed to improved detection rates and development in cancer screening and registry system [27, 29]. Furthermore, the incidence trends was the highest for 1–4 and < 1 year-old children with APC of 0.77% and 0.73% [3]. Ecologic studies suggest different risk factors for different types of cancers including genetic, race, environmental exposure to carcinogenic agents, life-style related factors [25, 29, 36]. More studies are warranted to investigate factors responsible for trends in these groups of children.

Tehran (including Alborz) was the only province with both high risk of cancer incidence and increasing trend. Tehran is the most populated province of the country with various races and higher air pollution. High risk and ascending trend of cancer incidence can indicate the clustering role of cancer risk factors in this province.

In the present study, 27% of the provinces exhibited ascending temporal trend in the cancer risk. The interesting point was ascending trend in provinces with low cancer incidence, which highlights the changes in cancer incidence pattern of these provinces and the need for serious research and intervention. The other noteworthy point is that high-risk provinces are located mainly in borders of the country. Numerous studies have highlighted the role of the distance to the center and socioeconomic conditions in cancer incidence and complications [31, 37]. More research is needed to examine if this could be the case in childhood cancer.

Lack of individual data on cancer cases and limited information on detailed childhood cancer and its types as a rare event halted further inferences in the current study. It would be helpful to consider risk factors in future ecological studies to factor in their spatial effects in risk assessments. Detection rates are expected to be improved due to establishments of cancer registries in the provinces. This may not leave a remarkable impact on our results as it is almost uniform over all provinces. Furthermore, the results are described in relative scale.

Conducting screening programs is not cost-effective for childhood cancer due to lower incidence rates in these age groups. On the other hand, early detection is fundamental in childhood cancer as treatments have promising results and the disease burden could be lowered. In this regard, our findings may be useful to prioritize provinces and regions with respect to research and budgeting in high risk areas and the provinces with steepest increasing trends.

## Conclusion

The risk pattern of cancer in Iranian children and adolescents seems to be different from that in adults and further research is imperative to identify high-risk groups and related risk factors. Guided screenings and expanding the health coverage could appropriately increase health system capacity and guarantee the access to effective childhood cancer detection, care, and treatment.

## Data Availability

Data were obtained from Iran MOHME. The datasets are available from the corresponding author on a reasonable request.
